# HIV-1 Tat C-mediated regulation of tumor necrosis factor receptor-associated factor-3 by microRNA 32 in human microglia

**DOI:** 10.1186/1742-2094-9-131

**Published:** 2012-06-18

**Authors:** Ritu Mishra, Chintan Chhatbar, Sunit Kumar Singh

**Affiliations:** 1 Laboratory of Neurovirology and Inflammation Biology, Centre for Cellular and Molecular Biology (CCMB), Council of Scientific and Industrial Research(CSIR), Uppal Road, Hyderabad, 500007, India

**Keywords:** HIV Tat protein, microRNA, HIV Neuroinflammation, HIV and miRNA, Tat and miRNA, HIV Tat and bystander effects, HIV Tat and gene regulation

## Abstract

**Background:**

HIV-1 Tat protein is known to be associated with neuroinflammation, a condition that develops in almost half of patients infected with HIV-1. HIV-1 Tat can alter glial neuroprotective functions, leading to neurotoxicity within the CNS. HIV-1 Tat is known to be secreted from productively infected cells and can affect neighboring uninfected cells by modulating cellular gene expression in a bystander fashion.

**Methods:**

We were interested to study whether exogenous exposure to HIV-1 Tat-C protein perturbs the microRNA (miRNA) expression profile of human microglial cells, leading to altered protein expression. We used protein expression and purification, miRNA overexpression, miRNA knockdown, transfection, site-directed mutagenesis, real-time PCR, luciferase assay and western blotting techniques to perform our study.

**Results:**

HIV-1 Tat-C treatment of human microglial cells resulted in a dose-dependent increase in miR-32 expression. We found that tumor necrosis factor-receptor–associated factor 3 TRAF3) is a direct target for miR-32, and overexpression of miR-32 in CHME3 cells decreased TRAF3 both at the mRNA and the protein level. Recovery of TRAF3 protein expression after transfection of anti-miR-32 and the results of the luciferase reporter assay provided direct evidence of TRAF3 regulation by miR-32. We found that the regulation of interferon regulatory factor 3 (IRF3) and IRF7 is controlled by cellular levels of TRAF3 protein in microglial cells, as after overexpression of miR-32 and application of anti-miR-32, expression levels of IRF3 and IRF7 were inversely regulated by expression levels of TRAF3. Thus, our results suggest a novel miRNA mediated mechanism for regulation of TRAF3 in human microglial cells exposed to HIV-1 Tat C protein. These results may help to elucidate the detrimental neuroinflammatory consequences of HIV-1 Tat C protein in bystander fashion.

**Conclusion:**

HIV-1 Tat protein can modulate TRAF3 expression through miRNA mediated pathway and can change the downstream expression of IRF3 and IRF7. This study demonstrates a novel mechanism of HIV-1 Tat C protein-mediated perturbation of miRNA, resulting in dysregulation of cellular TRAF3.

## Background

AIDS (acquired immunodeficiency syndrome) is caused by human immunodeficiency virus (HIV), which results in compromised immunity in the host. Although systemic immune cells such as CD4 positive T cells and macrophages are the prime targets for HIV infection, other cells, such as microglia and astrocytes, the resident cells of the CNS, are also reported to be productively infected by HIV [1]. Neuroinflammatory consequences of HIV infection into the CNS lead to complex neuropsychological and behavioral changes, collectively known as HIV-associated neurological disorder (HAND) [2]. Recent reports show that the prevalence of neurocognitive disorders has risen from 30% to 50% of patients infected with HIV [3]. HIV entry into the CNS is reported to take place during early acute infection [4] via infected macrophages, which cross the blood–brain barrier and transport the virus inside the CNS [5].

Antibodies against the HIV-1 transactivator of transcription (Tat) protein have been reported in the brains of patients with HIV encephalitis [6]. The HIV-1 Tat protein is essential for transactivation of viral and cellular genes [7], and has been reported to have a role in neuroinflammation, which ultimately leads to HIV-associated neurocognitive disorders [8].

HIV Tat has been shown to be the first protein expressed during HIV infection [9], and is capable of being actively released from the infected cells. Released Tat protein can be taken up by nearby infected and uninfected cells, thus affecting them in a bystander fashion [9]. In the CNS, microglia and astrocytes are often the first cells to respond to viral infection [10]. Microglia are resident macrophages of the CNS, and comprise about 10% of the total cell population of the brain [11]. Microglia protect the brain from pathogens, but overactivation of microglia can also result in inflammation, with subsequent damage to the neurons and hampering of brain functions [12,13]. The inflammatory mediators released by microglia strongly influence neurons and their ability to process information. Exogenous exposure of Tat mimics the extracellular release of HIV Tat protein from productively infected cells during HIV infection and acts as a model of the pathophysiological changes induced by Tat in bystander fashion. Neuropathological changes in the brains of patients infected with HIV have been attributed to various factors, including HIV Tat protein, but the exact mechanism of HIV Tat-mediated neuroinflammation is not well understood [14].

MicroRNAs (miRNAs) belong to a class of small non-coding RNAs ranging from 19 to 21 nucleotides in length, which are capable of regulating almost all cellular processes by suppressing translation of their target mRNAs [15,16]. Mature miRNAs are generated from longer primary RNA transcripts (pri-miRNAs), which are processed into shorter transcripts by the enzymes Drosha in the nucleus and Dicer in the cytoplasm [15,17]. Dysregulation of miRNA expression and function has been shown to be correlated with the altered levels of protein expression [18]. miRNA-mediated modulation of protein expression has been reported in various types of cancers and neurodegenerative diseases [19], and dysregulation of miRNAs has been reported in various neurological diseases. The *CYP2E1* gene, a cytochrome p450 isoform, is associated with Parkinson disease, and is regulated via miR-378 [20].

Changes in miRNA expression patterns have also been reported in HIV infection [19]. The HIV Tat protein has been reported to modulate neuronal functions via perturbations in the miRNA expression. Tat-mediated induction of miR-34a has been shown to downregulate specific genes [21], and this in turn leads to physiological changes in neurons, resulting in neuronal deregulation, neuronal loss, and consequently the development of HAND.

In this study, we examined whether HIV Tat protein can affect the levels of cellular proteins in uninfected cells in a bystander fashion by modulating miRNA expression patterns.

Tumor necrosis factor (TNF) receptor-associated factors (TRAFs) are intracellular adaptor proteins that bind to the cytoplasmic domain of TNF receptors and mediate downstream signaling [22]. The TRAF family is comprised of six proteins having a regulatory role in immune signaling. TRAF2 and TRAF5 have been reported to have positive functions, and TRAF3 and TRAF6 have both positive and negative regulatory functions in activation of the canonical nuclear factor kappa B (NF-κB) pathway [23,24]. TRAF3 is a major regulator of type I interferon (IFN) production and the innate antiviral response [25]. TRAF3 regulates both innate and adaptive immunity by modulating signaling mediated by various receptors such as Toll-like receptors, TNF receptors, and lymphotoxin-β receptor (LTBR) [12], and deficiency of TRAF3 has also been implicated in primary immunodeficiency diseases [26]. TRAF3 is a molecular switch that inhibits the LTBR-dependent activation of NF-κB1 [27], and has been reported to be capable of suppressing canonical NF-κB activation and downstream gene expression both *in vitro* and *in vivo*[28].

Degradative ubiquitination of TRAF3 after viral infection has been reported to be essential for virus-triggered interferon regulatory factor (IRF)3 activation and IFN induction [29]. Various studies have shown that inhibition of TRAF3 results in activation of both the canonical and non-canonical NF-κB activation pathways [27]. Collectively, these reports suggest that TRAF3 is an important suppressor of inflammatory responses through negative regulation of the canonical and noncanonical NF-κB pathways.

The mechanism of TRAF3 regulation in bystander cells through extracellularly secreted HIV Tat protein has not been reported to date. This study is the first, to our knowledge, to report that TRAF3 is targeted by miR-32. HIV-1 Tat C exposure leads to the upregulation of miR-32, which targets TRAF3 and regulates it post-transcriptionally. This finding demonstrates how extracellularly secreted viral proteins might modulate expression of their target genes through miRNA-mediated pathway in a bystander fashion.

## Experimental procedures

### Eukaryotic cell culture

Human microglial cells (CHME3) and HeLa cells were grown in DMEM (catlogue number 12100-046; Gibco-BRL, Gaithersburg, MD, USA) supplemented with 10% fetal bovine serum and 100 U of penicillin and 100 μg/ml streptomycin (10378016; Gibco-BRL). CEM-GFP cells (NIH-AIDS Reagent Program, Germantown, MD, USA) were cultured in RPMI 1640 (23400-021; Gibco-BRL) supplemented with 10% fetal bovine serum (16000-044; Gibco BRL), 2 mmol/l glutamine, 100 U/ml penicillin, and 100 U/ml streptomycin. All cells were incubated at 37°C in a humidified chamber supplemented with 5% CO_2_.

### Expression and purification of HIV tat protein

The *tat-c* gene was amplified from HIV-1 infected cells using the primer set listed in Table 1. The *tat-c* gene was cloned into the pET-21b bacterial expression vector. The His-tag was attached to Tat at the C terminal. To express recombinant Tat C protein, the *Escherichia coli* BL21 (DE3) strain was used. Protein production was induced for 3 hours, by adding isopropyl-β-D-thio-galactoside to give a final concentration of 1 mmol/l. Cell pellets were resuspended in 20 ml of lysis buffer (50 mmol/l phosphate buffer pH 7.9, 300 mmol/l KCl, 0.4 mmol/l EDTA, 10 mmol/l imidazole, 0.2 mmol/l phenylmethanesulfonyl fluoride, 1 mmol/l dithiothreitol, 10% glycerol, and 0.1% Triton-X 100). Cells were lysed by sonication, and proteins were purified by affinity chromatography using nickel-nitrilotriacetic acid (Ni-NTA) columns. Finally, Tat protein was eluted with phosphate buffer (pH 8.0) and 300 mmol/l imidazole, and was further purified and concentrated by centrifugal concentrators (Amicon MWCO 3 kDa; Millipore Corp., Billerica, MA, USA) and centrifugation was done at 4000 rpm, then resuspended in 30 mmol/l phosphate buffer, 70 mmol/l KCl, and 1 mmol/l DTT, and stored at −70°C. The identity of the recombinant Tat proteins was confirmed by western blot analysis using anti-Tat antibody (4138; NIH AIDS Research and Reference Reagent Program), and the level of endotoxin in recombinant Tat-C protein was checked by LAL assay (Lonza 50–647 U) in accordance with the manufacturer’s instructions.

**Table 1:** List of primers

**Gene**	**Direction**	**Primer sequence 5′→3′**
Tat –C	Forward	GGAATTCCATATGATGGAGCCAGTAGATCC
	Reverse	CCGCTCGAGATCGAATGGATCTGTCTTTG.
IFNB1	Forward	GCTCTCCTGTTGTGCTTCTCCAC
	Reverse	CAATAGTCTCATTCCAGCCAGTGC
IRF7	Forward	GCTGGACGTGACCATCATGTA
	Reverse	GGGCCGTATAGGAACGTGC
Beta-actin	Forward	TCATGAAGTGTGACGTGGAC
	Reverse	CAGGAGGAGCAATGATCTTGAT
TRAF3	Forward	GCGTGTCAAGAGAGCATCGTT
	Reverse	GCAGATGTCCCAGCATTAACT-3
IRF3	Forward	GTGGCCTGGGTGAACAAGAG
	Reverse	TGGAAGATTCCGAAATCCTCCT
MUT TRAF3 3′ UTR	Forward	CAACAAGATAAATGCTGTCAGAGAAGG3′
	Reverse	CCTTCTCTGACAGCATTTATCTTGTTG 3′

### Transactivation assay of HIV Tat protein

To test the functionality of the HIV Tat protein, we performed a transactivation assay using CEM-GFP cells (3655; NIH AIDS Research and Reference Reagent Program) containing a stably integrated green fluorescent protein (GFP) gene under the control of the HIV subtype B long terminal repeat (LTR). Cells were transfected with 5 μg/ml of purified Tat-C protein with a commercial transfection reagent (71281–3; ProteoJuice; Novagen/Millipore Corp., Billerica, MA, USA). GFP expression was visualized by fluorescence microscopy (Axio Imager; Carl Zeiss, Jena, Germany).

### Immunostaining and nuclear localization of HIV-1 Tat C protein

To track the localization of HIV-1 Tat C protein, CHME3 cells were seeded onto sterilized coverslips in six-well culture plates. After cells had achieved confluency, they were treated with 5 μg/ml Tat C protein. After 12 hours of treatment, cells were washed with PBS and fixed in 4% paraformaldehyde. Cells were permeabilized with 0.25% Triton X-100, and blocked with 3% BSA for 30 minutes, then incubated with the monoclonal Tat antibody (1:1000) overnight. Cells were washed with PBS and incubated with the secondary antibody conjugated to Alexa 488 (A-11008; Invitrogen Corp., Carlsbad, CA, USA). Cells were visualized under a fluorescence microscope (Axio Imager; Carl Zeiss, Jena, Germany).

### MicroRNA targets predictions

Bioinformatic prediction tools PicTar (http://pictar.mdc-berlin.de), Target Scan (version 5.2; http://www.targetscan.org) were used to identify the potential targets of miR-32. The miR-32 target binding sites in the 3′ untranslated region (UTR) of human TRAF3 transcripts were identified with TargetScan Human software as above.

### Tat treatment on human microglial cells

The human microglial cell line (CHME3) was grown till 75% confluency, and exposed to HIV Tat protein at 500 ng/ml concentration in serum-free media. After 24 hours of treatment, CHME3 cells were harvested for RNA isolation and protein studies.

### RNA isolation and microRNA assay

RNA isolation was performed with a commercial kit (217004; miRNeasy Mini Kit; Qiagen Inc., Valencia, CA, USA). The cDNA synthesis for miRNA was performed using miRNA specific primers and a commercial kit (4366596; TaqMan Reverse Transcription Kit; Applied Biosystems, Foster City, CA, USA), with the following settings: 16°C for 30 minutes, 42°C for 30 minutes, and 85°C for 5 minutes. miRNA assays were performed using quantitative (q)PCR with miRNA-specific Taqman probes and a master mix (4324018; Universal PCR Master Mix Applied Biosystems), using settings of 95°C for 10 minutes, followed by 40 cycles of 95°C for 15 seconds and 60°C for 60 seconds in a thermal cycler (ABI 7900; Applied Biosystems).

### Cell lysates and western blot analysis

RIPA buffer (150 mmol/l NaCl, 50 mmol/l Tris. HCL pH 7.0, 1% NP-40, 0.5% sodium deoxycholate, 0.1% SDS, and 1X protease inhibitor cocktail) was used to lyse cells. Protein concentrations were determined by the Bradford assay (500-0006; Bio-Rad Laboratories, Inc., Hercules, CA, USA). Equal amount of proteins were separated in a 12% polyacrylamide gel, and transferred onto polyvinylidene fluoride membrane at 100 V for 2 hours. The membrane was blocked in 5% skimmed milk powder prepared in Tris-buffered saline with Triton X-100 (TBS-T). Membranes were incubated overnight at 4°C with primary antibody (1:1000). After three washes of 10 minutes each with TBST, the horse-radish peroxidase-conjugated secondary antibody was applied for 45 minutes. Membranes were again washed in TBS-T three times and developed (34095; Super Signal Developing Reagent; Pierce Biotechnology Inc., Rockford, Illinois, USA).

### MicroRNA overexpression

Cells were seeded into six-well plates at 60% confluency 1 day before transfection. Transfection mixtures were prepared in commercial medium (11058–021; Opti-MEM; Invitrogen Corp.), and cells were kept in antibiotic-free medium during transfection. miR-32 overexpression was performed by transfecting the miRNA expression plasmid (SC400329; Origene Technologies, Rockville, MD, USA) using transfection reagent (11668–019; Lipofectamine 2000; Invitrogen Corp.). The empty vector was used as vehicle control. miR-32 overexpression was confirmed by qPCR using TaqMan probes specific to miR-32.

### Reverse transcription and real-time PCR

RNA was subjected to DNAse treatment (M0303L; New England Biolabs, Beverly, MA, USA) for 30 minutes at 37°C. cDNA synthesis was performed with reverse transcriptase (11904–18; Superscript II; Invitrogen) in accordance with the manufacturer’s protocol. PCR conditions were: 65°C for 5 minutes, 25°C for 10 minute, 42°C for 50 minutes, and 70°C for 10 minutes, and finally RNAse H treatment for 20 minutes at 37°C. SYBR Green (4309155; Applied Biosystems) was used for qPCR. The primer sequences used in the study for qPCR are listed in Table 1.

### Transfection with anti-microRNA (microRNA inhibitor)

CHME3 cells were transfected (Lipofectamine 2000; Invitrogen Corp.) with 100 pmol of anti-miR-32 (AM12584; Ambion, Foster City, CA, USA) and Cy3-labeled control anti-miR (AM17011; Ambion). After 48 hours of transfection, cells were pelleted for RNA isolation and protein lysate preparation. Transfection efficiency was assessed by visualizing the fluorescence of Cy3-labeled control anti-miR. Knockdown of miR-32 in anti-miR transfected cells was assessed using an miR-32 assay. TRAF3 protein expression in cells transfected with anti-miR-32 was analyzed using western blotting with anti-TRAF3 antibody (ab76147; Abcam, Cambridge, MA, USA).

### Luciferase reporter assay

HeLa cells were seeded in six-well plates and co-transfected (Lipofectamine 2000; Invitrogen Corp.) with luciferase reporter clones of TRAF3 3′ UTR and a miR-32-expressing plasmid. The TRAF3 3′ UTR construct in pMirTarget (SC206836; Origene Technologies) and miR-32 construct as pCMV-Mir (SC400329; Origene Technologies) were used. A mutation in the 3′ UTR of TRAF3 in miR-32 binding sites was created by deleting the TATT sequence at position 463 to 466 of the 3′ UTR using the primer set listed in Table 1. A site-directed mutagenesis kit (200518; Stratagene, La Jolla, CA, USA) was used for generating the deletion mutations. Both the wild-type (WT) and mutant 3′ UTR of TRAF3 were transfected along with miR-32 expression clones in HeLa cells. Cells were harvested after 24 hours of transfection for luciferase assays (E4030; Luciferase Assay Kit; Promega Corp., Madison, WI, USA) in accordance with the manufacturer’s protocol. A β-galactosidase assay (E2000; Promega Corp.) was used for normalization.

### Statistical analysis

Results are shown as the mean and standard error of the mean from three independent repeated experiments. Results are shown relative to controls in miR-32 assay. The level of significance (*P* values) between treated and untreated (control) groups was analyzed using the Student’s *t*-test, and *P* < 0.05 was considered significant.

## Results

### Expression and purification of tat protein

We could express the recombinant HIV-1 Tat C protein successfully by using the standard procedures as described in material and methods section. After the purification of HIV-1 Tat C protein; we used LAL assay to determine the endotoxin levels in the purified protein. The concentration of endotoxin was found to be 0.04 EU/μg of protein, which is greatly below the acceptable limits. To confirm the biological activity of the recombinant Tat protein, the transactivation assay was performed in CEM-GFP cells with stably integrated HIV-1 LTR. These cells showed enhanced transcription after 24 hours of Tat C transfection, as visualized by GFP expression (data not shown). Purified Tat C protein was found to be transcriptionally active as it significantly enhanced GFP expression through the transcription of HIV LTR.

### HIV-1 Tat C upregulates the expression level of microRNA 32

**Figure 1:**
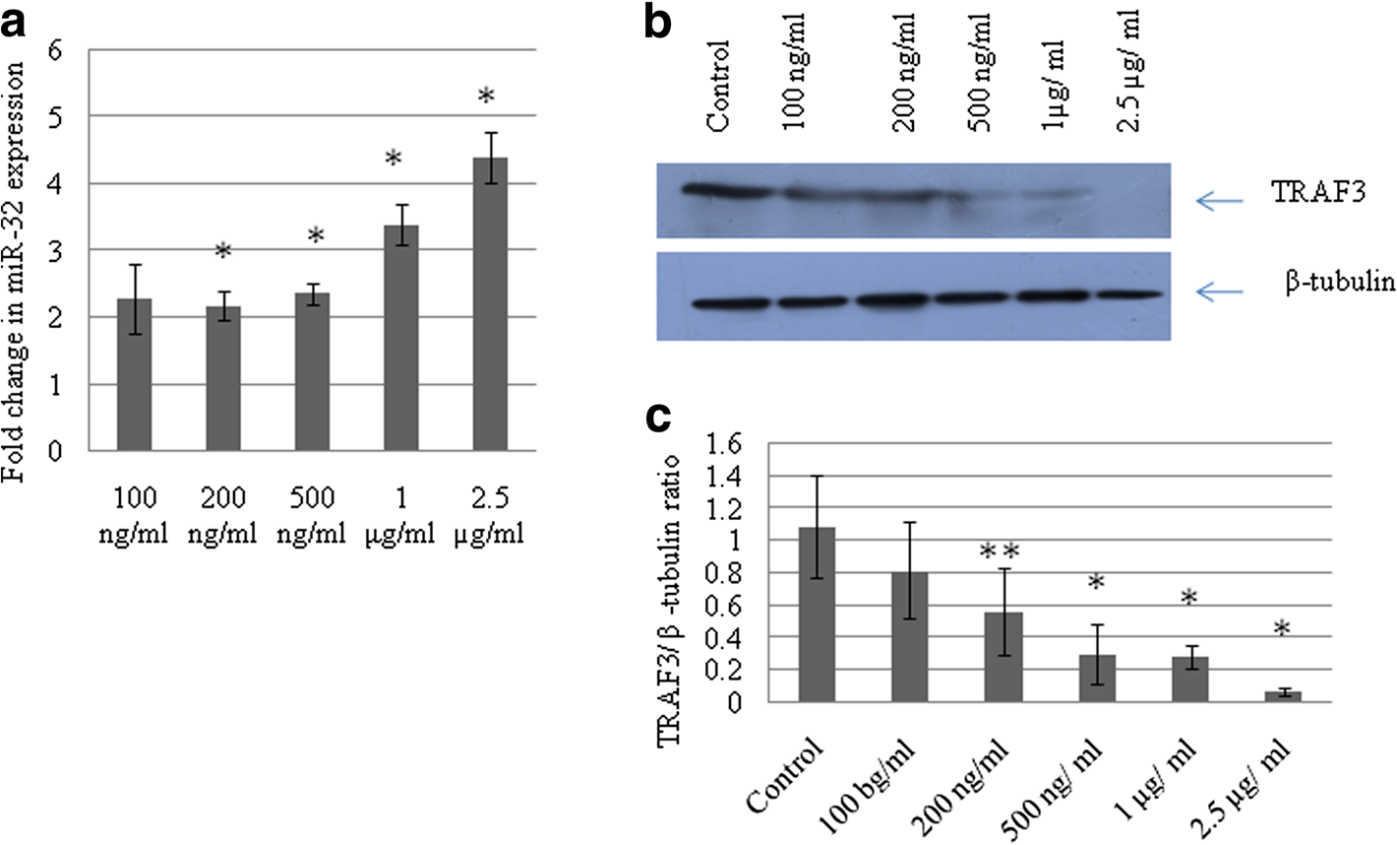
**Expression of miR-32 increases with Tat C treatment in a dose-dependent manner.(a)** CHME3 cells were treated with an increasing dose of HIV-1 Tat C protein. After 24 hours, cells were harvested for RNA isolation and protein lysate preparation. miR-32 assays were performed by quantitative PCR with TaqMan probes and primers specific for human miR-32. Data was normalized to the expression level of the small RNA, RNU24, and results are shown as fold change compared with untreated control. Changes in miR-32 expression level were significant (*P* ≤ 0.05). **(b)**, Western blot analysis for tumor necrosis factor receptor-associated factor 3(TRAF3) of the same samples treated with increasing concentrations of Tat C, showing a gradual reduction in TRAF3 protein expression. **(c)** Western blot image intensity was normalized to β-tubulin. All experiments were performed three times and are presented as mean ± SE. Changes in the level of expression of TRAF3 in response to increasing dose of Tat C were significant (**P ≤ 0.005, *P ≤ 0.05) compared with the untreated group.

HIV-1 Tat protein is known to activate the cellular genes, including expression of small RNAs [30]. CHME3 cells were treated with increasing dose of Tat-C protein to study the dose-dependent effect of Tat protein on miR-32 expression levels. Expression levels of miR-32 were assessed by qPCR using miR-32 specific TaqMan probes. A gradual increase in miR-32 expression levels was seen after Tat C treatment in a dose-dependent manner, with a 2.2-fold change in miR-32 expression seen in cells treated with 100 ng/ml of Tat C protein, and a 4.4-fold increase in cells treated 2.5 μg/ml of Tat C (Figure 1a).

### HIV-1 Tat C decreases tumor necrosis factor receptor-associated factor 3 levels in CHME3 cells

Using bioinformatic prediction tools (Pictar, TargetScan), we found that TRAF3 is a putative target of miR-32. To investigate whether changes in miR-32 expression affect the levels of TRAF3 (Figure 1A), we assessed the expression level of cellular TRAF3 protein in HIV-1 Tat C-treated CHME3 cells. TRAF3 protein level decreased sharply with the increase in Tat-C mediated upregulation of miR-32 (Figure 1b), suggesting that TRAF3 can be a direct target of miR-32 and that its expression can be modulated by changes in miR-32 expression.

**Figure 2:**
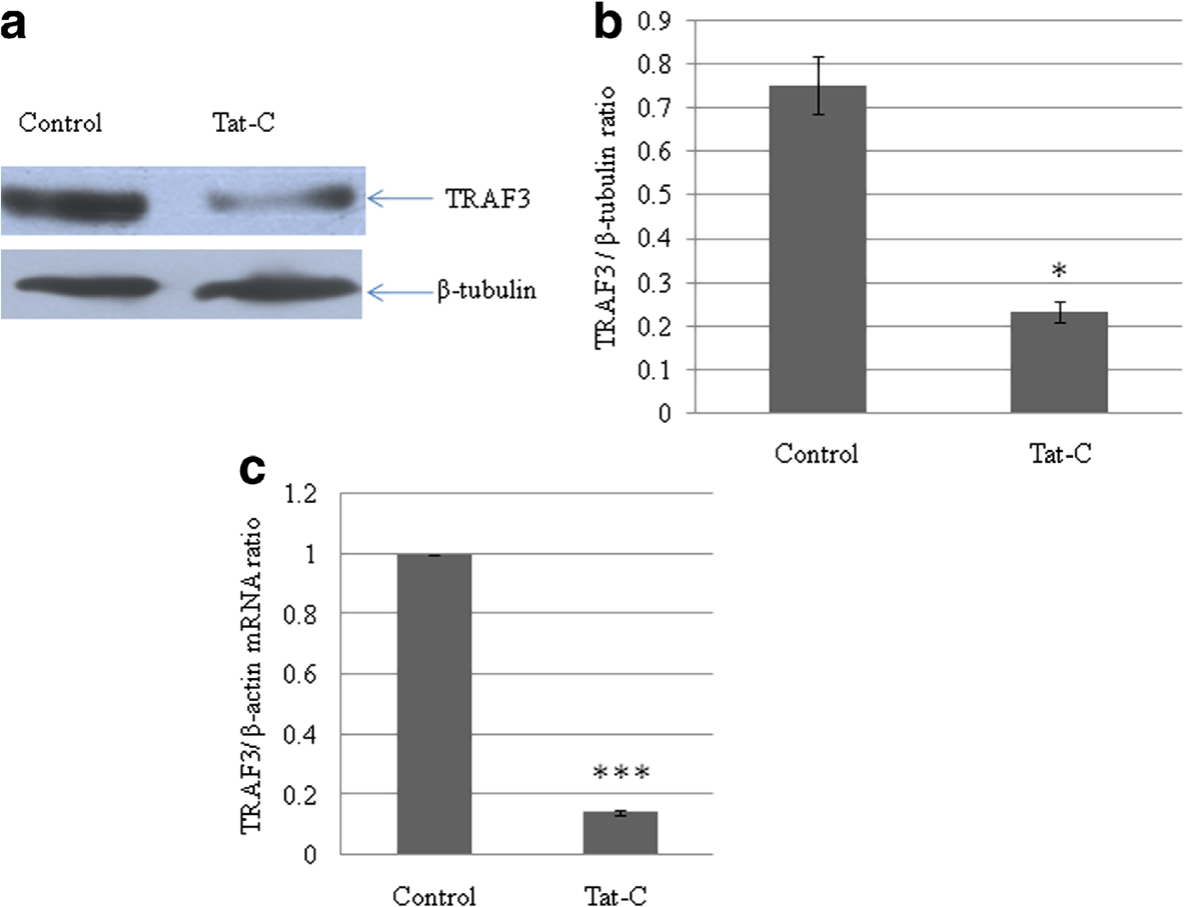
**HIV-1 Tat C protein downregulates tumor necrosis factor receptor-associated factor 3 (TRAF3) protein expression.(a)** Western blot analysis of TRAF3 in CHME3 cells exposed to HIV-1 Tat C protein. Treating CHME3 cells with 500 ng/ml Tat C significantly reduced the cellular TRAF3 protein level. **(b)** Densitometry analysis of TRAF3, normalized to β-tubulin image density. The change in TRAF3 expression level in the treated group versus the untreated control group was significant (*P ≤ 0.05). **(c)** Quantitative PCR analysis of TRAF3 in CHME3 cells exposed to HIV Tat C protein. The graph is representative of three independent experiments. All experiments were performed at least three times and data are presented as mean ± SE. **P ≤ 0.005.

In another experiment, CHME3 cells were exposed to Tat C (500 ng/ml) for 24 hours, and both mRNA and protein expression of TRAF3 decreased significantly (*P* < 0.05)(Figure 2A,C).

### No significant change in drosha and dicer levels after Tat C treatment

**Figure 3:**
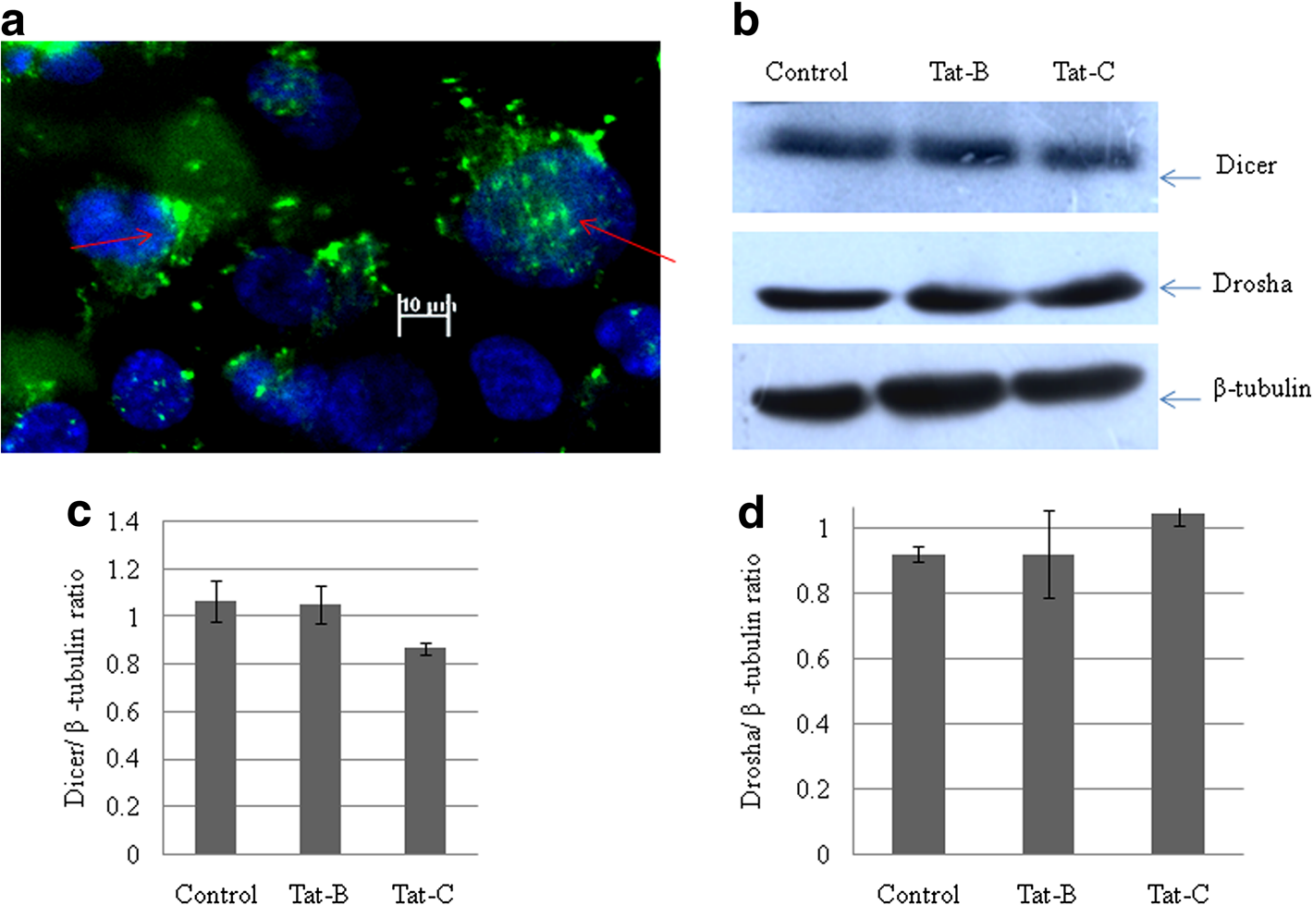
**HIV-1 Tat protein exerts no significant effect on the RNA interference machinery.(a)** Nuclear localization of Tat C protein in CHME3 cells exposed to recombinant HIV-1 Tat C protein. CHME3 cells were treated with Tat C protein at 5 μg per ml of culture media for 24 hours. Cells were then washed with PBS, fixed and stained with anti-Tat antibody, and visualized with Alexa 488-conjugated fluorescent antibody. **(b)** Western blot analysis for Dicer and Drosha in CHME 3 cells exposed to Tat C for 24 hours showed no significant change in the expression of either protein. β-tubulin was used as loading control for normalizing the image density. **(c)** Densitometry quantification for Drosha and Dicer enzyme normalized to β-tubulin. All experiments were performed three times, and data are presented as mean ± SE.

After exposing the CHME3 cells to the HIV-1 Tat C protein, the cellular localization of HIV-1 Tat C protein in CHME3 cells was visualized by immunostaining (Figure 3A). The localization study confirmed that recombinant HIV Tat C protein was internalized by cells and entered the nucleus (Figure 3a).

Viruses or viral proteins are known to influence the cellular RNA interference machinery. To assess whether changes in miR-32 expression levels after Tat C treatment involve the miRNA biogenesis machinery, we assessed the expression of Dicer and Drosha, and found no significant changes in their expression (Figure 3b), indicating that the Tat protein does not modulate the miRNA biogenesis machinery in CHME3 cells.

### microRNA 32 overexpression downregulates expression level of cellular tumor necrosis factor receptor-associated factor 3

**Figure 4:**
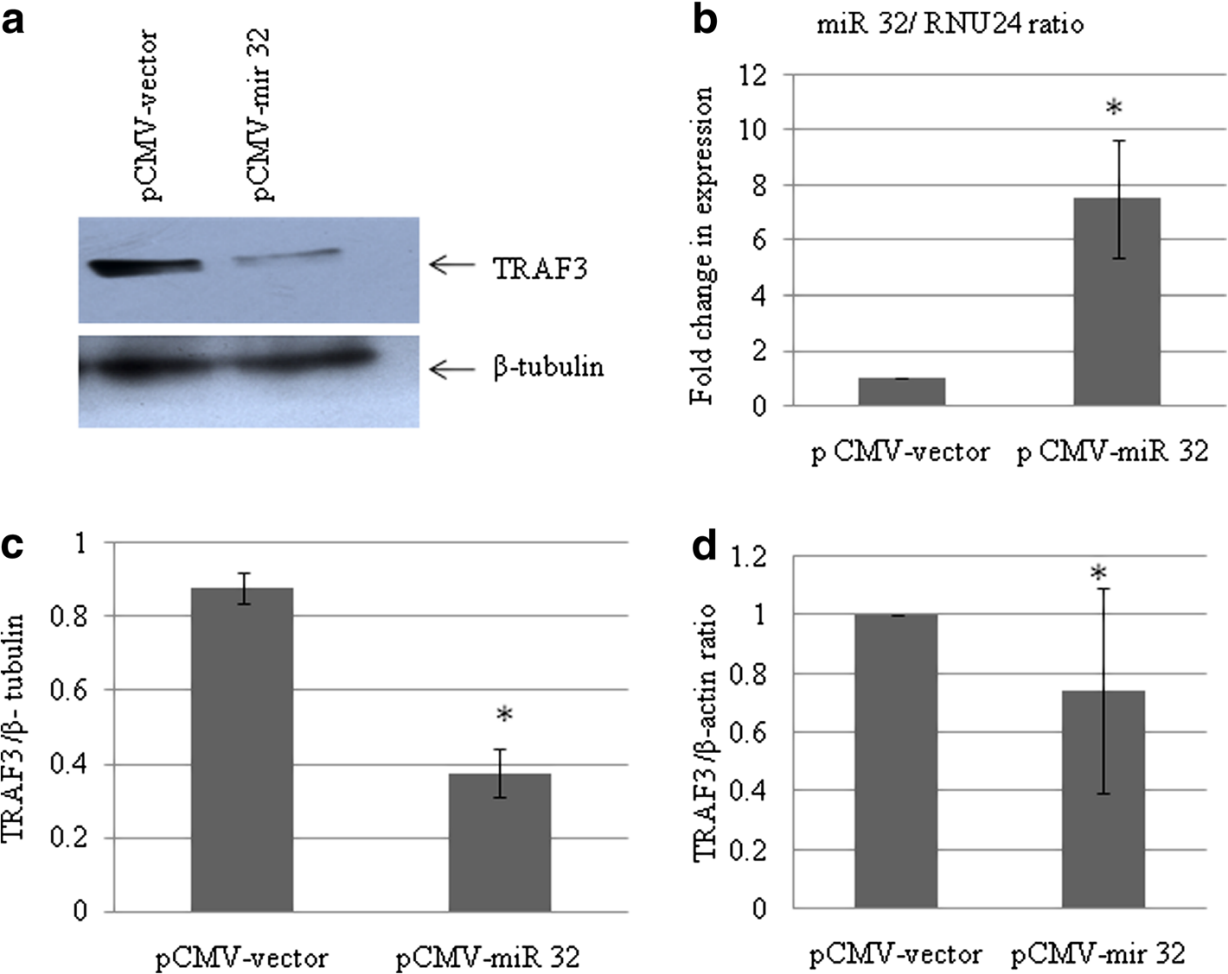
**Overexpresssion of miR-32 suppresses tumor necrosis factor receptor-associated factor 3(TRAF3) protein expression. (a)** Western blot analysis for TRAF3 in CHME3 cells after miR-32 overexpression. Plasmid pCMV-miR-32 was transfected into CHME3 cells. The empty vector was used as the negative control. Cell lysates were prepared after 24 hours of transfection, and western blot analysis was performed using anti-TRAF3 antibody. miR-32 overexpression significantly reduced both mRNA and protein levels of TRAF3 (*P* ≤ 0.05) (indicated by * in the transfected group) compared with empty vector. **(b)** Quantitative (q)PCR analysis of miR-32 overexpression in CHME3 cells, using TaqMan miR-32 assay. miR-32 expression was found to be 7.5-fold higher in miR-32-overexpressed cells. **(c)** Densitometry quantification of TRAF3 normalized to β-tubulin. **(d)** qPCR analysis for detection of changes in transcript level of TRAF3 after miR-32 overexpression in CHME3 cells. All experiments were performed at least three times and data are presented as mean ± SE.

A pCMV-miR-32 construct encoding miR-32 was transfected into CHME3 cells, and the expression level of miR-32 was determined after 24 hours of transfection. The level of miR-32 was 7.5-fold higher after transfection (*P* < 0.05) (Figure 4B). Therefore, we investigated expression of TRAF3 protein in miR-32-transfected CHME3 cells to establish a correlation between the expression level of miR-32 and that of TRAF3 protein. We found a reduction in protein expression and mRNA levels of TRAF3 after Tat C treatment, as well as reduction in the expression of TRAF3 protein as a consequence of miR-32 overexpression (Figure 4A). The overexpression of miR-32 (7.5-fold higher) led to a 60% reduction in the TRAF3 expression level compared with the cells transfected with empty vector. qPCR analysis confirmed the change in TRAF3 transcription after miR-32 overexpression in CHME3 cells (Figure 4D) Thus, miR-32 was found to target TRAF3, suggesting a direct link between expression of miR-32 and TRAF3.

### Anti-microRNA 32 rescues the expression level of tumor necrosis factor receptor-associated factor 3 protein

**Figure 5:**
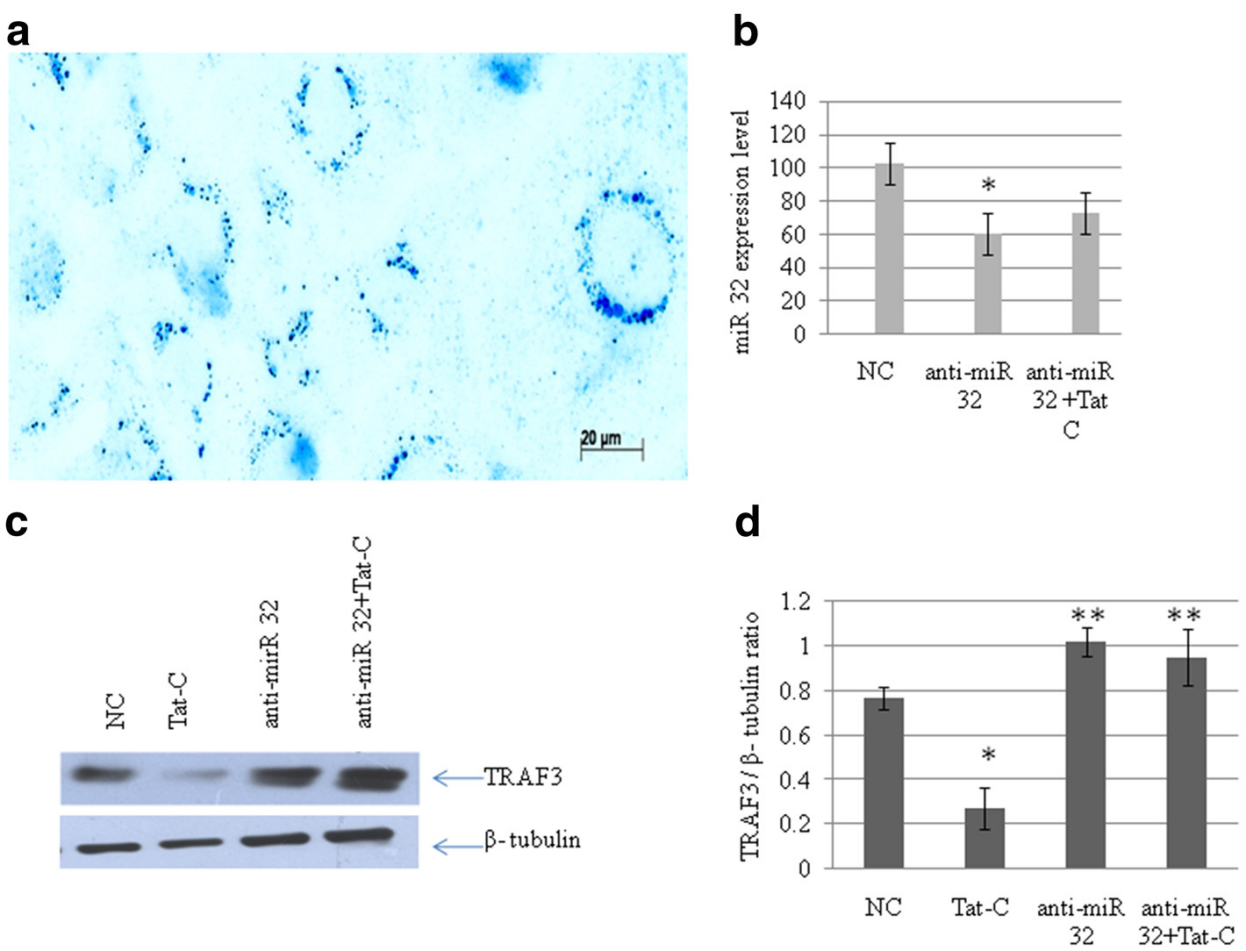
**Anti-miR-32 transfection rescues tumor necrosis factor receptor-associated factor 3 (TRAF3) protein expression in CHME3 cells. (a)** Transfection efficiency of anti-miR, by using Cy3-labeled anti-miR as negative control. **(b)** Quantitative (q)PCR analysis of cellular miR-32 level after anti-miR-32 transfection, to confirm the suppression of miR-32. The expression level of miR-32 decreased by 40% in cells transfected with anti-miR-32; compared to cells transfected with scrambled anti-miR negative control (*P ≤ 0.05). **(c)** Western blot analysis of TRAF3 in CHME3 cells after anti-miR-32 transfection, showing the recovery of TRAF3 expression level in cells treated with anti-miR-32 and anti-miR-32 plus Tat. Anti-miR-32 transfection was performed at a concentration of 100 pmol/l. After 24 hours of anti-miR-32 transfection, a set of transfected cells were treated with 500 ng/ml Tat C protein to augment the cellular expression level of miR-32. **(d)** Densitometry analysis of TRAF3 normalized to β-tubulin. There was a significant (**P ≤ 0.005) recovery of TRAF3 expression level.

This experiment was carried out to test the specificity of miR-32-mediated downregulation of TRAF3. CHME3 cells were transfected with anti-miR-32 (an miRNA inhibitor) along with a scrambled Cy3-labeled anti-miR-32 as negative control. The objective was to block the effects of miR-32 expression, which would confirm that these effects were due solely to the presence of miR-32. Anti-miR-32 was transfected into CHME3 cells, and this was followed by Tat C treatment. This procedure resulted in enhanced expression of TRAF3 (Figure 5c) and recovered the cellular TRAF3 level almost to that of control cells (Figure 5C,D). This demonstrates that cellular TRAF3 level can be recovered with the help of an antagonist against miR-32.

### microRNA 32 directly targets the 3′ untranslated region of tumor necrosis factor receptor-associated factor 3

**Figure 6:**
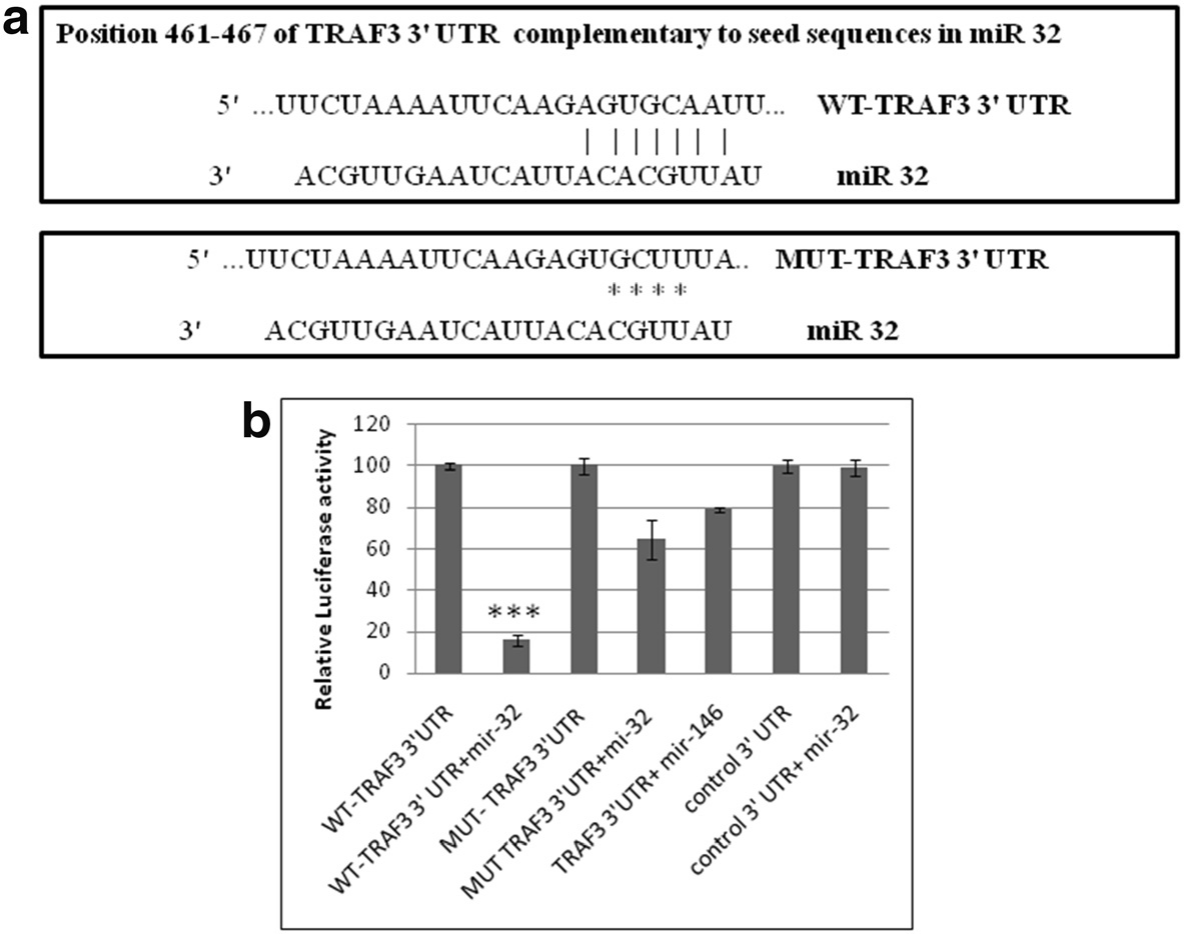
**miR-32 directly targets the 3′-UTR of tumor necrosis factor receptor-associated factor 3 (TRAF3).(a)** Seed sequence in miR-32 and complementary sequence in the 3′ UTR of TRAF3 mRNA showing seven-mer binding in wild-type (WT) TRAF3 3′ UTR. A deletion mutation of 4 base pairs in the 3′ UTR of TRAF3 was generated by site-directed mutagenesis. This alteration in the 3′ UTR sequence of TRAF3 abrogated the interaction of miR-32 and the 3′ UTR of TRAF3, resulting in translational derepression. **(b)** Luciferase assays were performed by transfecting HeLa cells with pCMV-β-gal (normalization control), WT TRAF3 3′ UTR and mutated (MUT) TRAF3 3′ UTR plasmids, along with pCMV-miR-32 plasmids. Normalized luciferase light units of control cells are presented as 100 units, and relative light units (RLU) of other treatments are shown accordingly. All experiments were performed three times and data are presented as mean ± SE (error bars). ***P ≤0.0005.

To test the mode of interaction between miR-32 and TRAF3, a luciferase assay was performed. In this experiment, luciferase reporter constructs was co-transfected with the miR-32 overexpression plasmid. In this reporter construct, the TRAF3 3′ UTR is flanked upstream by firefly luciferase coding sequences. Another construct was generated having deletions in the predicted binding site in the 3′ UTR of TRAF3 complementary to the seed region of miR-32. A deletion mutant was generated by site-directed mutageneis to modify the complementary sequence in the 3′ UTR of TRAF3 to abrogate the seven-mer match of the seed region of miR-32 and the TRAF3 3′ UTR. The empty construct without 3′ UTR of TRAF3, was simultaneously transfected with miR-32 overexpression plasmids as a negative control. A significant reduction of up to 80% in the luciferase activity was seen when the TRAF3 3′ UTR was cotransfected with miR-32 (*P* ≤ 0.0005). The TRAF3 3′ UTR mutant did not show a significant reduction in luciferase activity when transfected with miR-32. The TRAF3 3′UTR was transfected with a irrelevant miR-146 expression construct, which showed a much lower non-specific decrease in the luciferase activity (Figure 6b). The luciferase expression level of the control cells (transfected with TRAF3 3′UTR) was considered as 100%, and the reduction by miRNAs has been shown relative to this.

### Interferon regulatory factor (IRF)3 and IRF7 are perturbed by tat C treatment and microRNA 32 overexpression

**Figure 7:**
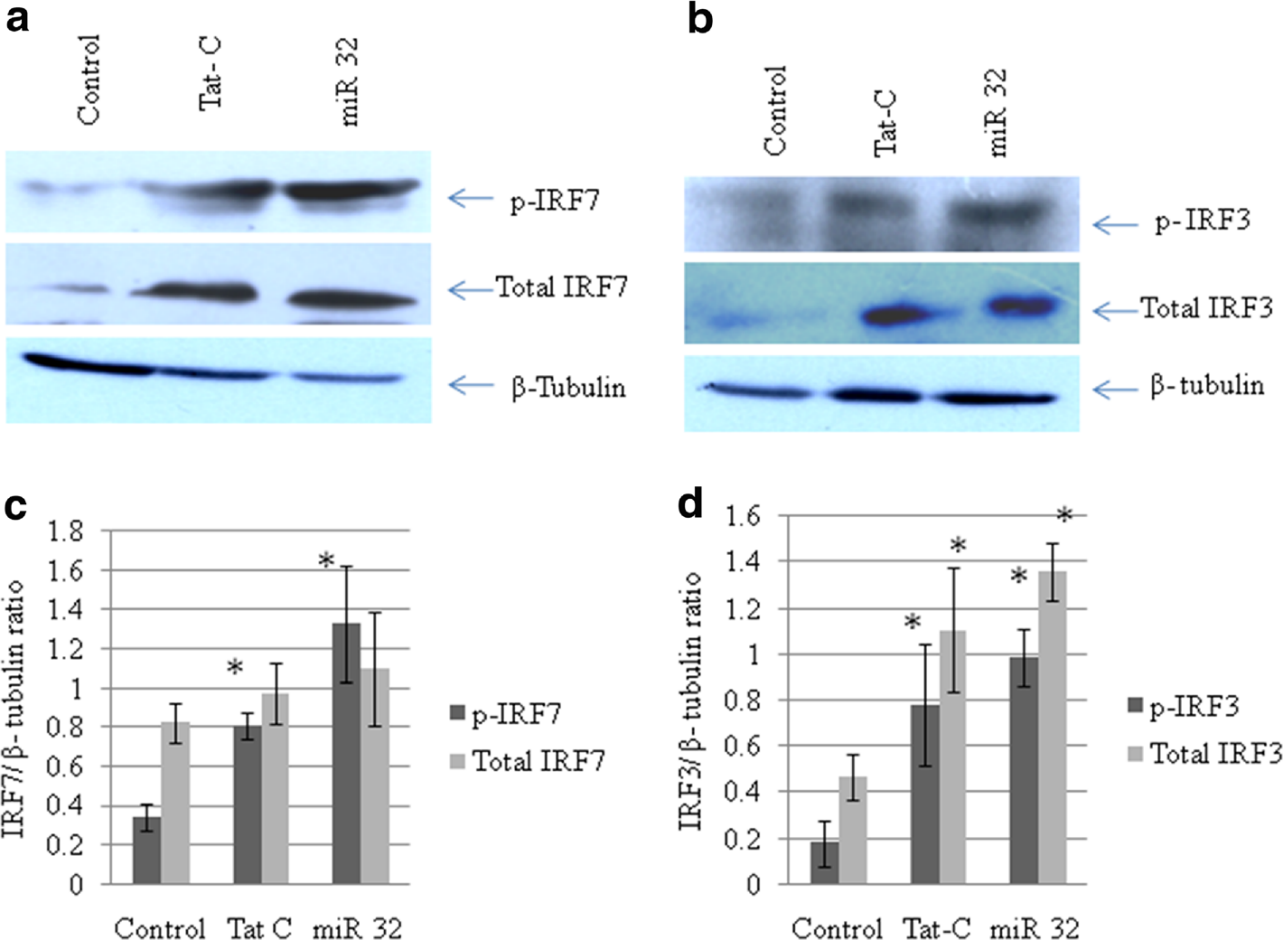
**Phosphorylated state and total interferon regulatory factor (IRF)3 and IRF7 changes in CHME3 cells treated with TatC and transfected with miR-32.(a)** Western blot analysis for phosphorylated (p)-IRF7 and total IRF7 in CHME3 cells after Tat C treatment and miR-32 transfection. Expression levels of both p-IRF7 and total IRF7 expression levels increased with both treatments. **(b)** Western blot analysis for p-IRF3 and total IRF3 in Tat C-treated and miR-32-transfected samples of CHME3 cells. Both total IRF3 and pIRF3 were upregulated with both treatments. Tat C at 500 ng/ml was applied for 24 hours. After 24 hours of miR-32 transfection, cells were harvested and lysed for protein samples and RNA isolation. **(c,d)** Densitometry analysis of p-IRF3, total IRF3, p-IRF7, and total IRF7 normalized to β-tubulin. All experiments were repeated three times, and data are presented as mean ± SE (error bars). Results were significant (*P ≤ 0.05).

IRF3 and IRF7 are constitutively located in the cytoplasm, and are phosphorylated and translocated to the nucleus in response to a stimulatory trigger. After translocation to the nucleus, they induce the expression of IFNs and other inflammatory cytokines. TRAF3 is regarded as a dual-mode repressor of the NF-κB pathway, having an important role in regulating the IFN response of cells during infections. Tat C treatment and miR-32 overexpression resulted in the same trend of activation of IRF3 and IRF7 (Figure 7). Western blot analysis showed higher levels of phosphorylated IRF3 and IRF7 in Tat C-treated cells (Figure 7A,B) and a similar trend was seen in cells overexpressing miR-32. Total protein levels of IRF3 and IRF7 were also found to be higher in Tat C treated and miR-32 overexpressing CHME3 cells (Figure 7). Downregulation of TRAF3 after Tat C treatment and miR-32 overexpression, and increased levels of phosphorylation of IRF3/7 suggest a negative regulatory role of TRAF3 in controlling IRF3 and IRF7 expression, which is in accordance with previously shown function of TRAF3 [27].

**Figure 8:**
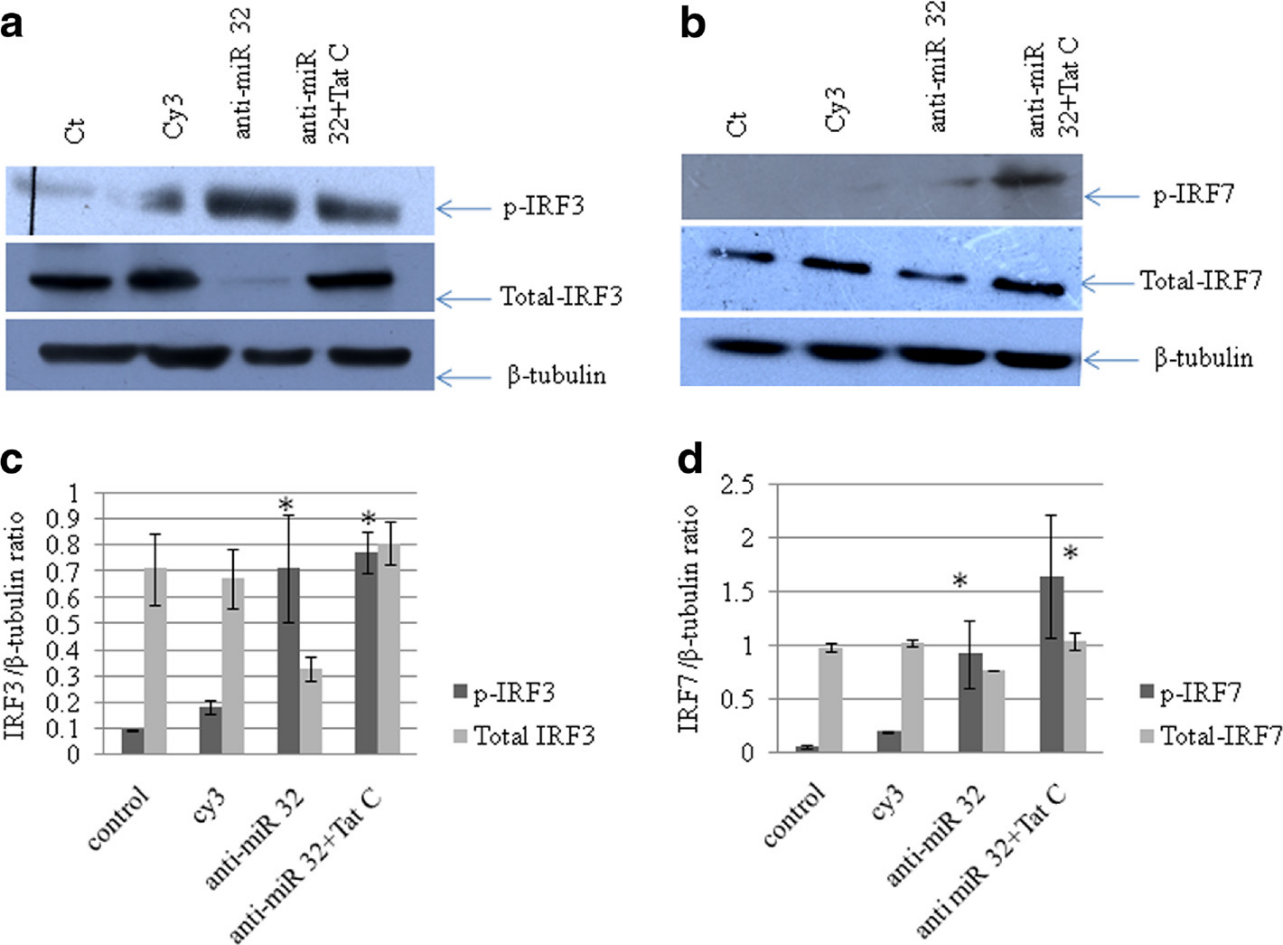
**Recovery of tumor necrosis factor receptor-associated factor 3 (TRAF3) expression by anti-miR-32 transfection suppresses expression levels of total interferon regulatory factor (IRF)3 and IRF7.(a)** CHME3 cells were transfected with Cy3-labeled control anti-miR,anti-miR-32 and anti-miR-32 plus Tat C treatment. Phosphorylated (p)IRF3 level increased after anti-miR-32 treatment, while the total IRF3 level was downregulated in anti-miR-32-transfected cells, showing a positive relationship between cellular TRAF3 level and activation of IRF3. **(b)** pIRF7 was increased after anti-miR-32 treatment and anti-miR-32 plus Tat C treatment, again showing a positive role of TRAF3 in IRF7 activation. Total IRF7 level was decreased in anti-miR-32-transfected cells showing that recovery of TRAF3 could modulate the transcription of IRF3and IRF7. **(c,d)** Densitometry analysis of pIRF3, pIRF7, total IRF3 and total IRF7 normalized to β-tubulin. Experiments were performed three times and data are presented as mean ± SE. Results were significant (*P ≤ 0.05).

The expression levels of total IRF3, IRF7 and pIRF3/7 in CHME3 cells transfected with anti-miR-32 were analyzed by western blotting. Expression levels of total IRF3 and IRF7 decreased (Figure 8A,B) in cells transfected with anti-miR-32, but phosphorylation of IRF3 and IRF7 increased compared with the scrambled anti-miR transfection (negative control).

**Figure 9:**
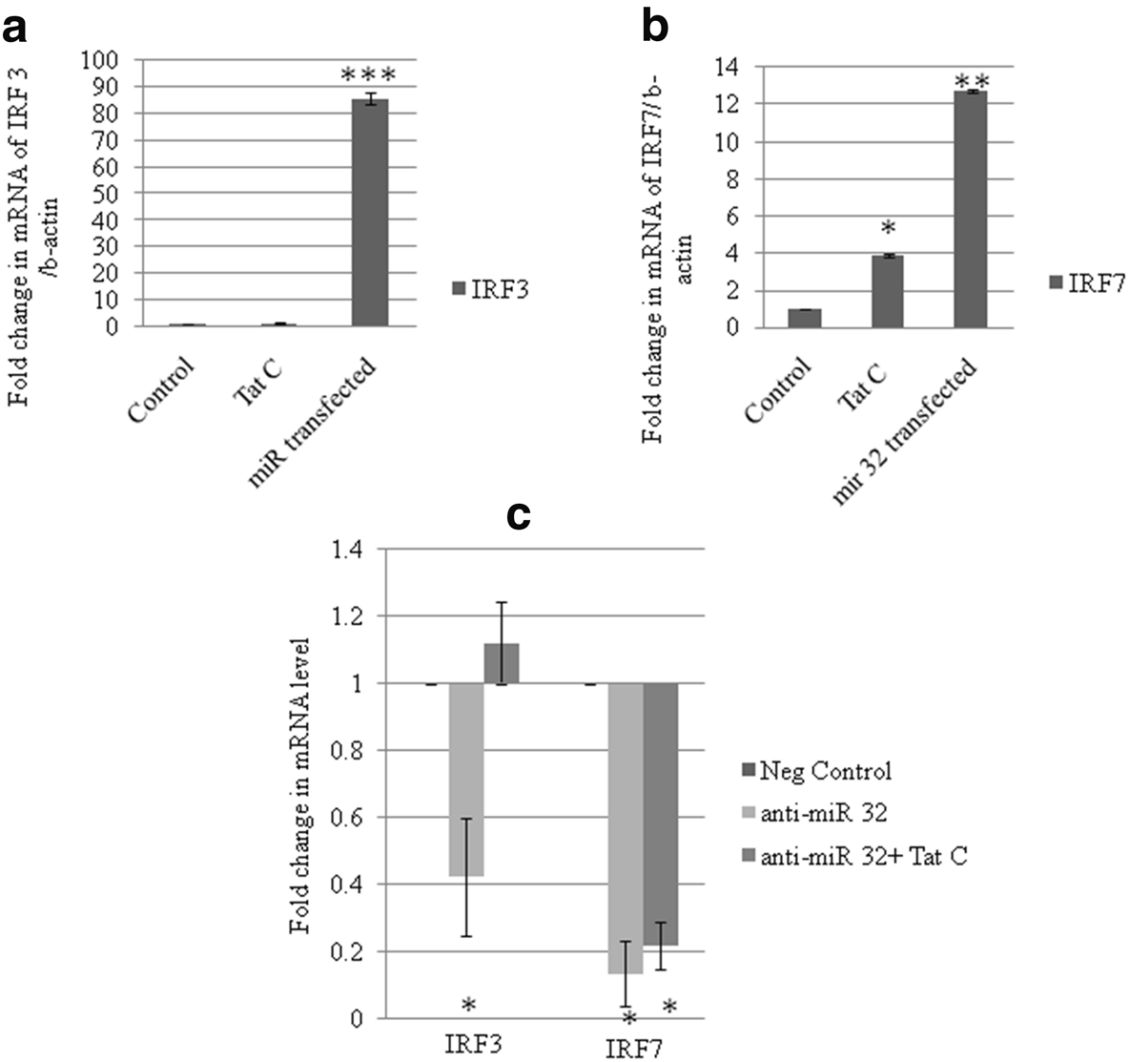
**Modulation of tumor necrosis factor receptor-associated factor 3 (TRAF3) protein level alters the interferon regulatory factor (IRF)3 and IRF7 mRNA level.(a,b)** CHME3 cells were treated with Tat C protein for 24 hours and transfected with miR-32, respectively. Relative fold changes in mRNA levels were determined for IRF3 and IRF7 using quantitative(q)PCR with SYBR green. As a consequence of Tat C treatment and miR-32 overexpression, the transcript expression levels of IRF3 and IRF7 increased. **(c)** After inhibiting the cellular miR-32 via application of anti-miR, the transcript level of both IRF3 and IRF7 was reduced. In cells treated with anti-miR-32 plus Tat C, the transcript levels of IRF3 and IRF7 were lower than those in control CHME3 cells. All experiments were repeated three times, and data are presented as mean ± SE. Relative change in IRF3 transcript in miR-32 transfected cells compared with empty vector were significant ***P ≤ 0.0005, **P ≤ 0.005 and *P ≤0.05 in respective graphs.

Both;the Tat C treatment and miR-32 overexpression increases the IRF3 and IRF7 expression at the transcript level (Figure 9A,B). The suppression of cellular miR-32 by using anti-miR-32, resulted in the reduced expression of both IRF3 and IRF7 at the transcript level (Figure 9C).

## Discussion

Microglia are brain resident macrophages that are involved in crucial regulatory processes of development and surveillance of the neural environment during injury, infection, and repair [11]. As the primary source for pro-inflammatory cytokines, microglial cells are implicated as pivotal mediators of neuroinflammation. Activated microglia leading to an inflammatory response has been reported in many models of neuroinflammation and neurotoxicity [11]. Viruses attempt to modulate the cellular environment using various strategies, changing the expression pattern of protein-coding genes and non-coding genes such as miRNAs [31]. Small RNAs have been reported to play an important role in neuroviral infections [21]. In the present study, we identified a novel mechanism by which HIV-1 Tat C protein can change the expression of specific genes in microglial cells by modulating expression of specific miRNAs. Minor variations in miRs can result in changes in the fine-tuning of cellular and biological processes [32,33]. Previous studies have shown that Tat induces molecular processes such as the proteasome-ubiquitin pathway for degradation and consequent downregulation of target protein [34]. In this study, we have for the first time identified a novel pathway by which HIV-1 Tat C protein can interfere with expression of the TRAF 3 gene in microglial cells by altering miRNA expression.

It has been shown that HIV Tat protein can be taken up by cells through the cellular endocytic machinery [35,36]. The immunostaining of recombinant HIV-1 Tat C-treated cells showed that HIV-1 Tat C protein moves to the nucleus and remains there to execute its transactivational functions. We also investigated the effect of HIV-1 Tat C protein on the miRNA biogenesis machinery because biogenesis of miRNAs begins in the nucleus. To study the effect on miRNA biogenesis, we examined the effect of HIV-1 Tat C protein on the major enzymes Drosha and Dicer, which play key roles in miRNA biogenesis, but we did not find any significant change in their expression.

Exposure of CHME3 cells to HIV-1 Tat C protein increased the expression level of cellular miR-32, accompanied by depletion of TRAF3 at protein level. The reciprocal relationship between increased miR-32 levels and reduced TRAF3 expression during Tat C exposure on CHME3 cells suggested an interaction between miR-32 and TRAF3. HIV Tat protein has been reported to induce the expression level of miR-128 in primary cortical neurons, which targets the 3′ UTR of the presynaptic protein SNAP25. This targeting leads to the suppression of SNAP25, whereas an anti-miR-128a antibody can restore SNAP25 expression [34]. In another study, Tat was reported to suppress the expression of the CYP2E1 protein in neurons, through the miR-1 mediated regulatory pathway. miR 1 was found to target the *Mef2A* gene; which in turn induced an miRNA cluster (miR-379 to 410) in neurons, which is important for dendritogenesis [37].

Other viral proteins have also been reported to suppress the cellular TRAF3 expression level [26]. Virus infection or exposure to double-stranded RNA has also been documented to decrease TRAF3 levels in a dose-dependent manner [38]. Both the mRNA and protein levels of the TRAF3 adaptor molecule have been reported to be downregulated in herpes simplex virus (HSV) infection [26]. Therefore, we designed this study to understand the mechanism by which extracellularly secreted HIV protein can affect gene expression in uninfected cells.

Bioinformatic databases predicted that a conserved recognition sequence for miR-32 was present in the 3′ UTR of TRAF3 (Figure 6A). miR-32 can regulate TRAF3 at the post-transcriptional level, through direct targeting of the TRAF3 3′ UTR. Target validation was performed using a reporter construct, having a firefly luciferase coding region fused with the 3′ UTR of TRAF3. By using a luciferase reporter assay, we showed that TRAF3 is indeed a direct target for miR-32. The targeting of miR-32 for the 3′ UTR of TRAF3 was specific as shown by the parallel experiment, in which irrelevant miR-146 was not able to affect the luciferase level.

The complementary sequence in the TRAF3 3′ UTR was modified by deleting four bases (TATT) at positions 463 to 466 to remove the binding site for miR-32. Luciferase expression was unaffected by using a mutated TRAF3 3′ UTR construct co-transfected with miR-32. This observation clearly supports the specificity of miR-32 binding to the TRAF3 3′ UTR in microglial cells. The complementary interaction between miR-32 and TRAF3 mRNA results in translational inhibition, leading to reduced expression of TRAF3 protein in CHME3 cells exposed to HIV-1 Tat C protein.

When miR-32 was overexpressed, the levels of TRAF3 mRNA and protein were downregulated in CHME3 cells (Figure 4). This effect can be simply attributed to enhanced cellular level of miR-32. The increase in miR-32 expression was confirmed by qPCR of miR-32, compared with CHME3 cells transfected with the empty vector (Figure 4b). The presence of complementary binding sites in the 3′ UTR of TRAF3 mRNA for miR-32 is most likely the way in which overexpression of miR-32 resulted in reduced expression of TRAF3.

To verify the specificity of miR-32 targeting to TRAF3 mRNA and blockage of translation, the level of cellular miR-32 was blocked by transfecting cells with anti-miR-32. This rescued the expression of TRAF3 protein and resulted in an enhanced level of TRAF 3, compared with control cells (Figure 5). This observation particularly revealed the inhibitory function of miR-32 on the expression of TRAF3. Blockage of cellular miR-32 by application of anti-miR-32 would have occupied the binding sites of mature miR-32, leaving the 3′ UTR of TRAF3 unrestricted, which resulted in enhanced protein expression. Additionally, when anti-miR-32-transfected cells were incubated with Tat C, the TRAF3 protein level was maintained at a higher level (Figure 5c). This suggests that anti-miR-32 could nullify the effect of Tat C-mediated upregulation of miR-32, and subsequent suppression of TRAF3 expression.

Modulation in TRAF3 expression could affect the immune functions of microglial cells. To study the downstream effect of TRAF3 reduction in altering the immune response, we studied the cellular expression levels of IRF3 and IRF7. IRFs are a family of transcription factors that play important roles in host defense systems [39]. In Tat C-treated and miR-32 transfected CHME3 cells, levels of phosphorylated IRF3 and IRF7 were higher. These results are in agreement with earlier reported functions of TRAF3 as a potent suppressor of inflammatory responses during overactivation in cells caused by any pathogenic insult [28]. In the present study, we found that the expression of TRAF3 decreased along with the increase in phosphorylated forms of IRF3 and IRF7 and levels of total IRF3 and total IRF7 after Tat C treatment or miR-32 overexpression. These findings show that changes in TRAF3 level affect expression levels of both pIRF3 and pIRF7, which can directly trigger the immune response, as well as the expression of total IRF3/7.

In another gain-of-function study of TRAF3, anti-miR-32 was transfected into CHME3 cells to assess the expression levels of phosphorylated forms of IRF3/7 and total IRF3/7. The phosphorylated IRF3/7 levels were found to be significantly higher, than those of the control (Figure 8), but total IRF3 and total IRF7 protein levels were decreased in anti-miR-32 transfected cells (Figure 8). These results are in accordance with the previously reported dual-mode repressor function of TRAF3, as adequate TRAF3 expression is required for the immune response [25] and for derepression of the alternative NF-κB pathway [27]. When TRAF3 protein levels are reduced in HIV-1 Tat C treated and miR-32-transfected cells, the repressive function of TRAF3 is removed, leading to activation of the non-canonical NF-κB pathway. As documented in previous studies, TRAF3 has been shown to bind constitutively to NIK (an essential activator of the alternative NFκB pathway) in unstimulated cells, and to block the activation of the non-canonical NF-κB pathway [40,41]. Our data suggest that reduction in TRAF3 level could release the NIK, which might lead to activation of the non-canonical NF-κB pathway. Constitutive activation of the NF-κB2 pathway has been reported as a consistent attribute of TRAF3 deficiency in multiple cell types [42]. However, TRAF3-deficient cells display only a partial reduction in IFN production after RNA virus infection and NF-κB activation [43].

Virus-triggered ubiquitylation of TRAF3 and TRAF6 by cIAP1 and cIAP2 has been reported as a necessary step for type I IFN induction and cellular antiviral response [29]. Degradative ubiquitination of TRAF3 has been reported to be necessary for the activation of mitogen-activated protein kinases and production of inflammatory cytokines [44].

A small interfering RNA-mediated depletion study of TRAF3 in DLD1 cells also showed a suppressive function of TRAF3 [27]. Decreased TRAF3 association with LTBR enhanced the recruitment of TRAF2 and IKK1 to LTBR-induced signaling complexes [27]. A reduced level of TRAF3 causes specific accumulation of a particular subset of NF-κB regulators, including key components of NF-κB2, such as p100, RelB, and NIK [27]. Another study showed that excess TRAF3 prevents recruitment of components (TRAF2 and IKK1) to receptor complexes necessary for NF-κB1 activation [45].

**Figure 10:**
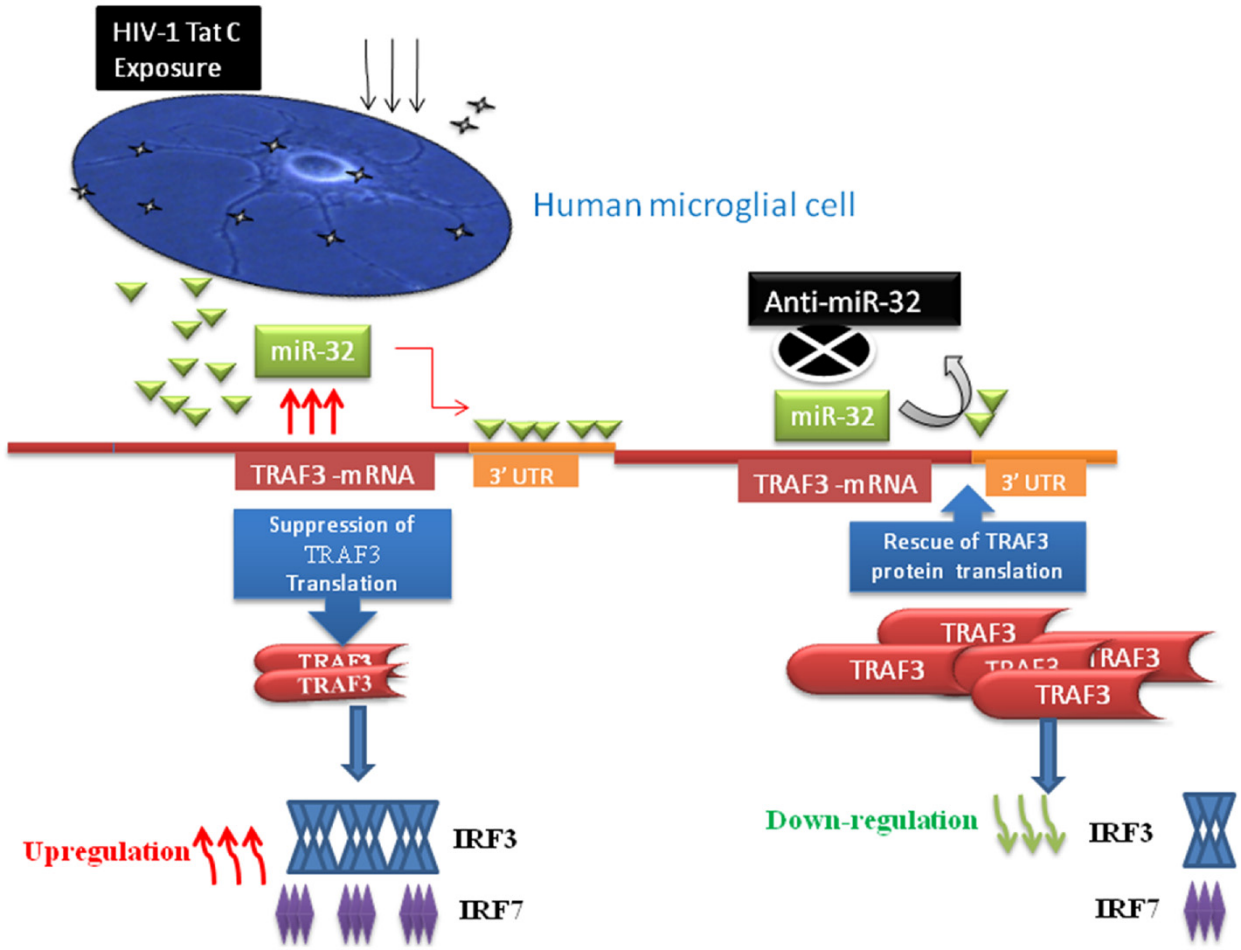
**Proposed model for HIV-1 Tat C-induced, miR-32-mediated post-transcriptional regulation of tumor necrosis factor receptor-associated factor 3 (TRAF3).** In response to HIV-1 Tat C exposure of human microglial cells, miR-32 was upregulated, consequently downregulating the protein level of TRAF3 post-transcriptionally by binding to its 3′ untranslated region. The miRNA inhibitor against miR-32, ant-miR-32, reduced the cellular level of miR-32 and rescued the expression level of TRAF3 protein. The cellular expression level of TRAF3 protein had an inverse relationship to the expression level of interferon regulatory factor (IRF)3/7 and this could perturb the expression of inflammatory genes in microglial cells after exposure to HIV-1 Tat C protein.

The changes in TRAF3 protein affected the total protein level of both IRF3 and IRF7 (Figure 5, Figure 7, Figure 8). Tat C-mediated miR-32 overexpression induced the expression levels of IRF3 and IRF7, indicating the correlation of reduced TRAF3 with increase in IRF3/7. Further, recovery of TRAF3 via anti-miR32 transfection resulted in decreased IRF3/7 at both at the mRNA and (Figure 9) and protein levels (Figure 8). Induction of type I IFNs requires coordinated and cooperative activation of the transcription factors IRF3/7 and NF-κB [46]. IFN-β is the early-phase IFN, which is primarily regulated by IRF3, whereas IFN-α is the late-phase IFN, activated by IRF7 through the STAT1 pathway [47]. Thus, HIV-1 Tat C protein can modulate the innate immune response by affecting the expression level of IRF3/7 operating via the TRAF3 expression level under the control of miR-32. In the present study,we suggest a new model (Figure 10) of miRNA-mediated regulation of the TRAF3 adaptor molecule in response to HIV-1 Tat C protein in microglial cells. Such miRNA-mediated dysregulation in TRAF3 expression might affect the immune activation of microglial cells, and thus might be one of several factors affecting neuroinflammation in patients infected with HIV. Further studies are required to understand the molecular basis of this regulation.

## Conclusion

In this study, we found that the expression of cellular TRAF3 protein in human microglial cells exposed to HIV-1 Tat C protein was regulated by cellular miR-32. The changes in expression levels of TRAF3 mediated by miR-32 resulted in changes in the expression pattern of cellular IRF3 and IRF7, which might lead to changes in interferon stimulatory genes. A well-orchestrated regulation of the innate immune response is very important to prevent damage caused by excessive or dysregulated activation of immune signaling factors. This study demonstrates the plausible mechanism of Tat-induced miRNA mediated dysregulation of the immune adaptor molecule TRAF3 in human microglial cells.

## Abbreviations

AIDS, Acquired immunodeficiency syndrome; BSA, Bovine serum albumin; CNS, Central nervous system; EDTA, ethylenediamene tetraacetic acid; GFP, Green fluorescent protein; HAND, Human immunodeficiency virus-associated neurological disorder; HIV, Human immunodeficiency virus; IFN, interferon; IRF, Interferon regulatory factor; LTBR, lymphotoxin-β receptor; LTR, Long terminal repeat; NF-κB, Nuclear factor kappa B; Ni-NTA, Nickel-nitrilotriacetic acid; PBS, Phosphate-buffered saline; qPCR, Quantitative polymerase chain reaction; SDS, sodium dodecyl sulfate; TBS-T, Tris-buffered saline with Triton X-100; TNF, Tumor necrosis factor; TRAF, Tumor necrosis factor receptor-associated factor; UTR, Untranslated region; WT, Wild type.

## Competing interests

The authors declare that they have no competing interests.

## Authors’ contributions

RM completed most of the experimental work related to cell culture, cloning, western blotting, transfection, quantitative real-time PCR and data analysis presented in this manuscript. CC helped in preparation of manuscript. SKS conceived the idea for the study, helped in designing the experiments, and critically supervised the complete study. All the authors read and approved the final revised manuscript.

## Authors’ information

RM is a recipient of a Senior Research Fellowship of the Council of Scientific and Industrial Research (CSIR), Government of India, and is pursuing her PhD studies at the Centre for Cellular and Molecular Biology (CCMB), Hyderabad. CC is a recipient of a Senior Research Fellowship of CSIR, Government of of India and is pursuing his PhD work at CCMB, Hyderabad. SKS is a neurovirologist, currently working as a scientist at the CCMB, Hyderabad. His research group is involved in research areas of HIV and vector-borne viral infections.

## References

[B1] Thompson KA Cherry CL Bell JE McLean CA Brain cell reservoirs of latent virus in presymptomatic HIV-infected individuals Am J Pathol 2011 179 1623 1629 10.1016/j.ajpath.2011.06.039 21871429 PMC3181362

[B2] Heaton RK Clifford DB Franklin DR Woods SP Ake C Vaida F Ellis RJ Letendre SL Marcotte TD Atkinson JH HIV-associated neurocognitive disorders persist in the era of potent antiretroviral therapy: CHARTER Study Neurology 2010 75 2087 2096 10.1212/WNL.0b013e318200d727 21135382 PMC2995535

[B3] Sharma D Bhattacharya J Cellular & molecular basis of HIV-associated neuropathogenesis Indian J Med Res 2009 129 637 651 19692743

[B4] Kaul M Zheng J Okamoto S Gendelman HE Lipton SA HIV-1 infection and AIDS: consequences for the central nervous system Cell Death Differ 2005 12 Suppl 1 878 892 15832177 10.1038/sj.cdd.4401623

[B5] Sanders VJ Mehta AP White MG Achim CL A murine model of HIV encephalitis: xenotransplantation of HIV-infected human neuroglia into SCID mouse brain Neuropathol Appl Neurobiol 1998 24 461 467 10.1046/j.1365-2990.1998.00145.x 9888156

[B6] Del Valle L Croul S Morgello S Amini S Rappaport J Khalili K Detection of HIV-1 Tat and JCV capsid protein, VP1, in AIDS brain with progressive multifocal leukoencephalopathy J Neurovirol 2000 6 221 228 10.3109/13550280009015824 10878711

[B7] Ju SM Song HY Lee JA Lee SJ Choi SY Park J Extracellular HIV-1 Tat up-regulates expression of matrix metalloproteinase-9 via a MAPK-NF-kappaB dependent pathway in human astrocytes Exp Mol Med 2009 41 86 93 10.3858/emm.2009.41.2.011 19287189 PMC2679334

[B8] Deshmane SL Mukerjee R Fan S Sawaya BE High-performance capillary electrophoresis for determining HIV-1 Tat protein in neurons PLoS One 2011 6 e16148 10.1371/journal.pone.0016148 21249135 PMC3017553

[B9] Li W Li G Steiner J Nath A Role of Tat protein in HIV neuropathogenesis Neurotox Res 2009 16 205 220 10.1007/s12640-009-9047-8 19526283

[B10] Lewis SD Butchi NB Khaleduzzaman M Morgan TW Du M Pourciau S Baker DG Akira S Peterson KE Toll-like receptor 7 is not necessary for retroviral neuropathogenesis but does contribute to virus-induced neuroinflammation J Neurovirol 2008 14 492 502 10.1080/13550280802345723 19016073

[B11] Kraft AD Harry GJ Features of microglia and neuroinflammation relevant to environmental exposure and neurotoxicity Int J Environ Res Public Health 2011 8 2980 3018 10.3390/ijerph8072980 21845170 PMC3155341

[B12] Antony JM Grooming and growing with microglia Sci Signal 2010 3 jc8 10.1126/scisignal.3147jc8 21062990

[B13] Chugh P Fan S Planelles V Maggirwar SB Dewhurst S Kim B Infection of human immunodeficiency virus and intracellular viral Tat protein exert a pro-survival effect in a human microglial cell line J Mol Biol 2007 366 67 81 10.1016/j.jmb.2006.11.011 17157319 PMC7127718

[B14] Hudson L Liu J Nath A Jones M Raghavan R Narayan O Male D Everall I Detection of the human immunodeficiency virus regulatory protein tat in CNS tissues J Neurovirol 2000 6 145 155 10.3109/13550280009013158 10822328

[B15] Singh SK miRNAs: from neurogeneration to neurodegeneration Pharmacogenomics 2007 8 971 978 10.2217/14622416.8.8.971 17716230

[B16] Fang JH Zhou HC Zeng C Yang J Liu Y Huang X Zhang JP Guan XY Zhuang SM MicroRNA-29b suppresses tumor angiogenesis, invasion, and metastasis by regulating matrix metalloproteinase 2 expression Hepatology 2011 54 1729 1740 10.1002/hep.24577 21793034

[B17] Lee Y Ahn C Han J Choi H Kim J Yim J Lee J Provost P Radmark O Kim S Kim VN The nuclear RNase III Drosha initiates microRNA processing Nature 2003 425 415 419 10.1038/nature01957 14508493

[B18] O’Connell RM Rao DS Chaudhuri AA Baltimore D Physiological and pathological roles for microRNAs in the immune system Nat Rev Immunol 2010 10 111 122 10.1038/nri2708 20098459

[B19] Eacker SM Dawson TM Dawson VL Understanding microRNAs in neurodegeneration Nat Rev Neurosci 2009 10 837 841 19904280 10.1038/nrn2726 PMC4120241

[B20] Shahabi HN Westberg L Melke J Hakansson A Belin AC Sydow O Olson L Holmberg B Nissbrandt H Cytochrome P450 2E1 gene polymorphisms/haplotypes and Parkinson’s disease in a Swedish population J Neural Transm 2009 116 567 573 10.1007/s00702-009-0221-1 19381774

[B21] Chang JR Mukerjee R Bagashev A Del Valle L Chabrashvili T Hawkins BJ He JJ Sawaya BE HIV-1 Tat protein promotes neuronal dysfunction through disruption of microRNAs J Biol Chem 2011 286 41125 41134 10.1074/jbc.M111.268466 21956116 PMC3220514

[B22] Ely KR Kodandapani R Wu S Protein-protein interactions in TRAF3 Adv Exp Med Biol 2007 597 114 121 10.1007/978-0-387-70630-6_9 17633021

[B23] Saitoh T Nakayama M Nakano H Yagita H Yamamoto N Yamaoka S TWEAK induces NF-kappaB2 p100 processing and long lasting NF-kappaB activation J Biol Chem 2003 278 36005 36012 10.1074/jbc.M304266200 12840022

[B24] Hacker H Tseng PH Karin M Expanding TRAF function: TRAF3 as a tri-faced immune regulator Nat Rev Immunol 2011 11 457 468 10.1038/nri2998 21660053

[B25] Oganesyan G Saha SK Guo B He JQ Shahangian A Zarnegar B Perry A Cheng G Critical role of TRAF3 in the Toll-like receptor-dependent and -independent antiviral response Nature 2006 439 208 211 10.1038/nature04374 16306936

[B26] Perez de Diego R Sancho-Shimizu V Lorenzo L Puel A Plancoulaine S Picard C Herman M Cardon A Durandy A Bustamante J Human TRAF3 adaptor molecule deficiency leads to impaired Toll-like receptor 3 response and susceptibility to herpes simplex encephalitis Immunity 2010 33 400 411 10.1016/j.immuni.2010.08.014 20832341 PMC2946444

[B27] Bista P Zeng W Ryan S Bailly V Browning JL Lukashev ME TRAF3 controls activation of the canonical and alternative NFkappaB by the lymphotoxin beta receptor J Biol Chem 2010 285 12971 12978 10.1074/jbc.M109.076091 20185819 PMC2857099

[B28] Zarnegar B Yamazaki S He JQ Cheng G Control of canonical NF-kappaB activation through the NIK-IKK complex pathway Proc Natl Acad Sci U S A 2008 105 3503 3508 10.1073/pnas.0707959105 18292232 PMC2265190

[B29] Mao AP Li S Zhong B Li Y Yan J Li Q Teng C Shu HB Virus-triggered ubiquitination of TRAF3/6 by cIAP1/2 is essential for induction of interferon-beta (IFN-beta) and cellular antiviral response J Biol Chem 2010 285 9470 9476 10.1074/jbc.M109.071043 20097753 PMC2843197

[B30] Romani B Engelbrecht S Glashoff RH Functions of Tat: the versatile protein of human immunodeficiency virus type 1 J Gen Virol 2010 91 1 12 10.1099/vir.0.016303-0 19812265

[B31] Boss IW Renne R Viral miRNAs and immune evasion Biochim Biophys Acta 2011 1809 708 714 10.1016/j.bbagrm.2011.06.012 21757042 PMC3864029

[B32] Hornstein E Shomron N Canalization of development by microRNAs Nat Genet 2006 38 Suppl S20 S24 16736020 10.1038/ng1803

[B33] Cao X Pfaff SL Gage FH A functional study of miR-124 in the developing neural tube Genes Dev 2007 21 531 536 10.1101/gad.1519207 17344415 PMC1820895

[B34] Eletto D Russo G Passiatore G Del Valle L Giordano A Khalili K Gualco E Peruzzi F Inhibition of SNAP25 expression by HIV-1 Tat involves the activity of mir-128a J Cell Physiol 2008 216 764 770 10.1002/jcp.21452 18381601 PMC2662126

[B35] Frankel AD Pabo CO Cellular uptake of the tat protein from human immunodeficiency virus Cell 1988 55 1189 1193 10.1016/0092-8674(88)90263-2 2849510

[B36] Cardarelli F Serresi M Albanese A Bizzarri R Beltram F Quantitative analysis of Tat peptide binding to import carriers reveals unconventional nuclear transport properties J Biol Chem 2011 286 12292 12299 10.1074/jbc.M110.203083 21321119 PMC3069432

[B37] Fiore R Khudayberdiev S Christensen M Siegel G Flavell SW Kim TK Greenberg ME Schratt G Mef2-mediated transcription of the miR379-410 cluster regulates activity-dependent dendritogenesis by fine-tuning Pumilio2 protein levels EMBO J 2009 28 697 710 10.1038/emboj.2009.10 19197241 PMC2647767

[B38] Nakhaei P Mesplede T Solis M Sun Q Zhao T Yang L Chuang TH Ware CF Lin R Hiscott J The E3 ubiquitin ligase Triad3A negatively regulates the RIG-I/MAVS signaling pathway by targeting TRAF3 for degradation PLoS Pathog 2009 5 e1000650 10.1371/journal.ppat.1000650 19893624 PMC2766052

[B39] Wang L Toomey NL Diaz LA Walker G Ramos JC Barber GN Ning S Oncogenic IRFs Provide a Survival Advantage for Epstein-Barr Virus- or Human T-Cell Leukemia Virus Type 1-Transformed Cells through Induction of BIC Expression J Virol 2011 85 8328 8337 10.1128/JVI.00570-11 21680528 PMC3147954

[B40] Xiao G Fong A Sun SC Induction of p100 processing by NF-kappaB-inducing kinase involves docking IkappaB kinase alpha (IKKalpha) to p100 and IKKalpha-mediated phosphorylation J Biol Chem 2004 279 30099 30105 10.1074/jbc.M401428200 15140882

[B41] Xiao G Harhaj EW Sun SC NF-kappaB-inducing kinase regulates the processing of NF-kappaB2 p100 Mol Cell 2001 7 401 409 10.1016/S1097-2765(01)00187-3 11239468

[B42] Hildebrand JM Yi Z Buchta CM Poovassery J Stunz LL Bishop GA Roles of tumor necrosis factor receptor associated factor 3 (TRAF3) and TRAF5 in immune cell functions Immunol Rev 2011 244 55 74 10.1111/j.1600-065X.2011.01055.x 22017431 PMC3202299

[B43] Tang ED Wang CY TRAF5 is a downstream target of MAVS in antiviral innate immune signaling PLoS One 2010 5 e9172 10.1371/journal.pone.0009172 20161788 PMC2820086

[B44] Tseng PH Matsuzawa A Zhang W Mino T Vignali DA Karin M Different modes of ubiquitination of the adaptor TRAF3 selectively activate the expression of type I interferons and proinflammatory cytokines Nat Immunol 2010 11 70 75 10.1038/ni.1819 19898473 PMC2872790

[B45] He L Grammer AC Wu X Lipsky PE TRAF3 forms heterotrimers with TRAF2 and modulates its ability to mediate NF-{kappa}B activation J Biol Chem 2004 279 55855 55865 10.1074/jbc.M407284200 15383523

[B46] Matsuzawa A Tseng PH Vallabhapurapu S Luo JL Zhang W Wang H Vignali DA Gallagher E Karin M Essential cytoplasmic translocation of a cytokine receptor-assembled signaling complex Science 2008 321 663 668 10.1126/science.1157340 18635759 PMC2669719

[B47] Sun F Zhang YB Liu TK Shi J Wang B Gui JF Fish MITA serves as a mediator for distinct fish IFN gene activation dependent on IRF3 or IRF7 J Immunol 2011 187 2531 2539 10.4049/jimmunol.1100642 21795596

